# Cholesterol Hydroxylating Cytochrome P450 46A1: From Mechanisms of Action to Clinical Applications

**DOI:** 10.3389/fnagi.2021.696778

**Published:** 2021-07-08

**Authors:** Irina A. Pikuleva, Nathalie Cartier

**Affiliations:** ^1^Department of Ophthalmology and Visual Sciences, Case Western Reserve University, Cleveland, OH, United States; ^2^NeuroGenCell, Paris Brain Institute, ICM, Inserm U 1127, CNRS UMR 7225, Sorbonne Université, Paris, France

**Keywords:** CYP46A1, cholesterol, 24-hydroxycholesterol, brain, phosphorylation, plasma membranes, lipid rafts, neurodegenerative diseases

## Abstract

Cholesterol, an essential component of the brain, and its local metabolism are involved in many neurodegenerative diseases. The blood-brain barrier is impermeable to cholesterol; hence, cholesterol homeostasis in the central nervous system represents a balance between *in situ* biosynthesis and elimination. Cytochrome P450 46A1 (CYP46A1), a central nervous system-specific enzyme, converts cholesterol to 24-hydroxycholesterol, which can freely cross the blood-brain barrier and be degraded in the liver. By the dual action of initiating cholesterol efflux and activating the cholesterol synthesis pathway, CYP46A1 is the key enzyme that ensures brain cholesterol turnover. In humans and mouse models, CYP46A1 activity is altered in Alzheimer’s and Huntington’s diseases, spinocerebellar ataxias, glioblastoma, and autism spectrum disorders. In mouse models, modulations of CYP46A1 activity mitigate the manifestations of Alzheimer’s, Huntington’s, Nieman-Pick type C, and Machao-Joseph (spinocerebellar ataxia type 3) diseases as well as amyotrophic lateral sclerosis, epilepsy, glioblastoma, and prion infection. Animal studies revealed that the CYP46A1 activity effects are not limited to cholesterol maintenance but also involve critical cellular pathways, like gene transcription, endocytosis, misfolded protein clearance, vesicular transport, and synaptic transmission. How CYP46A1 can exert central control of such essential brain functions is a pressing question under investigation. The potential therapeutic role of CYP46A1, demonstrated in numerous models of brain disorders, is currently being evaluated in early clinical trials. This review summarizes the past 70 years of research that has led to the identification of CYP46A1 and brain cholesterol homeostasis as powerful therapeutic targets for severe pathologies of the CNS.

## Introduction

Cholesterol is an essential component of the brain constituting 22 and 27.5% of dry weight in human gray and white matter, respectively (Quarles et al., 1999). In gray matter, cholesterol is mainly found in cellular membranes of glial cells and neurons (∼30% of total brain cholesterol as determined in mice). In white matter, cholesterol is at an abundance in myelin sheaths (∼70% of total brain cholesterol as determined in mice) (Dietschy and Turley, 2001, 2004), which represent greatly extended and modified plasma membranes of oligodendrocytes wrapped around axons in a spiral fashion (Quarles et al., 1999). As a membrane constituent, cholesterol influences biophysical (e.g., ordering, fluidity, and permeability) and biological (e.g., function of membrane-bound proteins and lipid rafts) properties of the membranes (Yeagle, 1991; Simons and Ehehalt, 2002), and thereby is involved in important brain processes such as transmission of electrical impulses along axons, synaptogenesis, and synaptic function (Mauch et al., 2001; Pfrieger, 2003; Segatto et al., 2014; Egawa et al., 2016). In addition, cholesterol is used in the brain for production of neurosteroids and oxysterols, the biologically active compounds involved in different brain regulatory and signaling pathways (Mellon and Griffin, 2002; Moutinho et al., 2016; Sun et al., 2016b; Björkhem and Diczfalusy, 2020).

**FIGURE 1:**
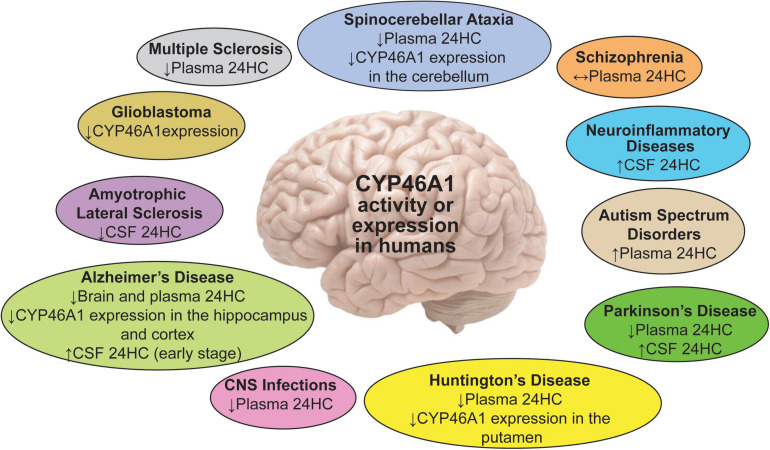
A summary of the 24HC or CYP46A1 measurements in human plasma, CSF and brain regions in different brain disorders. Upwards (↑), downwards (↓), and left-right (↔) arrows indicate increase, decrease, and no change.

The majority of cerebral cholesterol is synthesized locally, as the blood-brain barrier prevents cholesterol exchange with the systemic circulation (Dietschy and Turley, 2001). Similarly, cerebral cholesterol removal is also mainly enzymatic with the major route being 24-hydroxylation catalyzed by cytochrome P450 46A1 or CYP46A1 (Bjorkhem et al., 1998; Lund et al., 2003). Studies in humans and mouse models detected changes in CYP46A1 expression or activity in a number of neurodegenerative diseases [Alzheimer’s (AD), Huntington’s (HD), Nieman-Pick type C, spinocerebellar ataxias (Sca), multiple sclerosis (MS), and amyotrophic lateral sclerosis (ALS)] as well as other brain disorders (epilepsy, autism, Rett syndrome, glioblastoma, and prion infection) (Figure 1; Loftus et al., 1997; Boussicault et al., 2016; Lopez et al., 2017; Grayaa et al., 2018; Lütjohann et al., 2018; Vejux et al., 2018; Han et al., 2019; Huang et al., 2019; Nobrega et al., 2019; Sodero, 2020; Steriade et al., 2020; Ali et al., 2021). Hence, CYP46A1 has emerged as a therapeutic target for these diseases because of its key role in cerebral cholesterol elimination.

This review will first summarize the early seminal research, which created conceptual and pre-clinical bases for the on-going clinical pharmacological and gene therapy strategies developed to use CYP46A1 as a therapeutic target to treat neurodegenerative diseases. Then, the current clinical trials evaluating CYP46A1 modulations will be described, after which a discussion of the most recent studies aimed at understanding how this single enzyme, CYP46A1, can represent a common therapeutic target for various brain diseases. Finally, a list of pressing questions will be offered from the viewpoints of a biochemist (IAP), who pioneered the investigation of CYP46A1 pharmacological modulations and is now identifying brain processes affected by CYP46A1 activity, and a gene therapy neuroscientist (NC), aiming to ultimately bring this therapeutic target to patients affected with severe neurodegenerative diseases. More comprehensive reviews on CYP46A1, oxysterols, and the links between CYP46A1, cholesterol homeostasis in the brain, and neurological disorders can be found elsewhere (Russell et al., 2009; Moutinho et al., 2016; Bjorkhem et al., 2019; Loera-Valencia et al., 2019; Petrov and Pikuleva, 2019; Choi and Finlay, 2020; Griffiths and Wang, 2020; Sodero, 2020).

## 24-Hydroxycholesterol and CYP46A1

24-Hydroxycholesterol (24HC) was first detected in the brain of different mammalian species almost 70 years ago and was called cerebrosterol because of its abundance in this organ (Di Frisco et al., 1953; Ercoli et al., 1953). Then, about 50 years ago, the enzymatic origin of 24HC was established–from brain cholesterol and the reaction catalyzed by an endoplasmic reticulum (ER) mixed function oxidase or a P450 enzyme (Dhar et al., 1973; Lin and Smith, 1974). Later, the monooxygenase mechanism of the 24HC formation was also shown in a different study (Bjorkhem et al., 1997). The investigations in 1996–1998 revealed that the brain contains ∼80% of 24HC in the human body, and that there is a continuous flux of 24HC from the brain in the systemic circulation. Most of the circulating 24HC was found to be of cerebral origin and accounted for at least 75% of cholesterol removal and turnover in human brain; the liver represented the major site for subsequent 24HC biotransformations (Bjorkhem et al., 1998). Plasma 24HC levels were determined to markedly depend on age and be ∼5 times lower in the sixth decade of human life than in the first decade of life (Lutjohann et al., 1996).

cDNAs for mouse and human cholesterol 24-hydroxylases were cloned in 1999 and demonstrated to convert cholesterol to 24HC when transfected into cultured cells (Lund et al., 1999). In both species, RNA and protein expression were predominantly confined to the brain, where the signals were detected in some, but not all, neurons of the cortex, hippocampus, dentate gyrus, and thalamus. The amino acid sequences of mouse and human cholesterol 24-hydroxylases shared a high 95% identity and suggested protein localization to the ER. The identified enzymes were the members of a new cytochrome P450 family, 46, subsequently designated as cytochromes P450 46A1 or CYP46A1. Ontogeny measurements in mice and humans documented a marked discrepancy between the 24HC levels in the serum and brain. In mice, serum 24HC levels declined with age after postnatal days 12–15, whereas the brain 24HC levels increased with age and were closely matched by an increase in the CYP46A1 expression. In human brain (the frontal lobe), CYP46A1 expression was also low at young age (before 1 year) and much higher between the ages of 1.5 and 72 years (Lund et al., 1999). CYP46A1 was found to be ectopically expressed in brain astrocytes in AD and other cell types in mouse models of MS and traumatic brain injury (Bogdanovic et al., 2001; Brown et al., 2004; Teunissen et al., 2007; Cartagena et al., 2008). Still, CYP46A1 selectivity in neuronal expression and ER localization remained valid for normal conditions (Ramirez et al., 2008). Recently, the spatial 24HC distribution in mouse brain was determined and found to reflect local *Cyp46a1* expression (Yutuc et al., 2020).

*Cyp46a1^–/–^* mice were generated in 2003 and revealed to have a tight coupling between cholesterol elimination and biosynthesis from the brain as their brain cholesterol levels were unchanged because of a compensatory decrease in the brain cholesterol biosynthesis (Lund et al., 2003). The CYP46A1 contribution to cholesterol removal and turnover in mouse brain was determined and was shown to be at least 40–50% (Lund et al., 2003). Detailed studies of CYP46A1 enzymatic properties were initiated in 2003 when human CYP46A1 was heterologously expressed in *E. coli* and purified. In the reconstituted system *in vitro*, purified CYP46A1 converted cholesterol to 24HC and further hydroxylated 24HC to 24, 25- and 24, 27-dihydroxycholesterols. The rate of the CYP46A1-mediated cholesterol hydroxylation was slow when compared with other cholesterol hydroxylases (CYP7A1, CYP11A1, and CYP27A1) (Mast et al., 2003), but consistent with slow cholesterol turnover in human (∼9.1 years) and mouse (∼0.7 year) brains (Bjorkhem et al., 1998; Dietschy and Turley, 2001). Besides cholesterol, other compounds (some marketed drugs and neurosteroids) were metabolized by CYP46A1 *in vitro*, thus suggesting that the P450 active site is plastic and can accommodate molecules of different sizes, shapes, and polarities. Later, this plasticity of the enzyme active site was confirmed by crystal structures of CYP46A1 with and without the substrate cholesterol sulfate and in complex with seven different pharmaceuticals: the antidepressants tranylcypromine and fluvoxamine; the antifungals voriconazole, clotrimazole, and posaconazole; the anticonvulsant thioperamide; and the anticancer drug bicalutamide (Mast et al., 2008, 2010, 2012, 2013a,b). The biochemical and biophysical characterizations of purified CYP46A1 finalized the 24HC-CYP46A1 link and created the basis for subsequent P450 investigations as a pharmacologic target.

## 24HC in Plasma and Cerebrospinal Fluid (CSF) as a Potential Biomarker in Neurological Diseases

The potential diagnostic power of plasma 24HC for onset and progression of several neurodegenerative diseases has been the subject of multiple studies with fascinating, yet sometimes inconsistent, results (Figure 1). These inconsistencies were likely due to multiple factors: heterogeneity of patient populations, disease stage, brain atrophy associated with advanced disease, specific kinetics of disease evolution, complex interactions involving brain metabolic balance, function of the blood-brain barrier, and inflammatory components. Indeed, at an early disease stage, cell loss from myelin breakdown and neurodegeneration may lead to a release of both cholesterol and 24HC, thus increasing cholesterol availability to CYP46A1. Accordingly, brain cholesterol turnover and plasma 24HC levels may be increased. Conversely, at an advanced disease stage, there is significant brain atrophy and a reduced number of the CYP46A-containing cells along with a decreased neuronal expression of CYP46A1 (see below). Hence, at this stage, brain cholesterol turnover and plasma 24HC levels may decrease. During intermediate stages, opposite effects on the brain cholesterol turnover may overlap and compensate each other, with no change in the plasma 24HC levels (Leoni and Caccia, 2013a; Björkhem and Diczfalusy, 2020).

A summary of the reproducible findings is presented in Figure 1 with the most compelling data coming from the studies in patients with AD and HD. More recently, investigations of PD, ALS, and MS as well brain trauma, neonatal hypoxia, and glioblastoma have reinforced the interest for 24HC as a biomarker of disease progression and highlighted the role of CYP46A1 in this process or even disease genesis.

### Plasma 24HC in Neurological Diseases

In AD, an increase, decrease or no change in plasma 24HC levels were documented in affected patients (Bretillon et al., 2000; Lutjohann et al., 2000; Papassotiropoulos et al., 2002; Schonknecht et al., 2002; Heverin et al., 2004; Kolsch et al., 2004; Zuliani et al., 2011; Popp et al., 2012; Leoni and Caccia, 2013a; Li et al., 2018). This inconsistency was explained by the disease stage, as different criteria were used for the disease stage determination and patient enrollment. Also, multiple other factors were found to affect plasma 24HC levels (Leoni and Caccia, 2013a; Björkhem and Diczfalusy, 2020). These factors included inflammation, dysfunction of the blood-brain barrier, ectopic CYP46A1 expression in astrocytes, and medications that altered the whole body and brain cholesterol metabolism. These factors further limited the diagnostic power of 24HC for AD (Leoni and Caccia, 2013a). Recently, a systematic oxysterol profiling in the brain of patients with different stages of AD provided important insight into oxysterol changes during the disease progression (Testa et al., 2016). In the early stages of AD, the levels of 24HC in the frontal and occipital cortex were only slightly below those of controls, while at the later AD stages, the 24HC levels were markedly and significantly decreased (40%), thus raising a possibility that this concentration change may play an important role in the end-phase of the disease.

In HD, decreases in plasma 24HC levels were reported at all stages, but were normal in premanifest patients. This result was explained to reflect disease burden, loss of neurons, and degree of structural atrophy (Leoni et al., 2008; Leoni and Caccia, 2013b; Leoni et al., 2013a). Also, the finding of the same reduction in plasma 24HC across different HD stages, despite a progressive decrease in the caudate volume (Leoni et al., 2008), suggested that perhaps there was an uncoupling of the 24HC production in the HD-affected brains and in the cholesterol biosynthesis, which was also reduced. As a result, cholesterol content in the caudate in the HD brain was elevated (Del Toro et al., 2010), and more cholesterol became available to CYP46A1 for the 24HC production to compensate for the caudate volume decrease.

In MS, a recent study showed that serum 24HC levels were lower in primary progressive and older relapsing remitting patients. Furthermore, the serum levels of cholesterol precursors (lathosterol, lanosterol, desmosterol) were decreased in all clinical subtypes (Teunissen et al., 2003; van de Kraats et al., 2014; Mukhopadhyay et al., 2017).

In PD, initial studies investigating plasma 24HC levels showed inconsistent results (Lee et al., 2009; Bjorkhem et al., 2013; Huang et al., 2019). In the cohorts of up to 25 disease-affected participants, both lower and unchanged plasma oxysterol levels were found relative to the controls (Lee et al., 2009; Bjorkhem et al., 2013; Di Natale et al., 2018). However, in a recent investigation with a sample size of 35 PD patients and a controlled analysis for potential confounders, a robust evidence was provided that higher levels of 24HC are inversely associated with the risk of the disease (Huang et al., 2019).

Severe CNS infections were also associated with a reduction in plasma 24HC levels (Bretillon et al., 2000; Leoni and Caccia, 2013b). Conversely, children with autism spectrum disorders were shown to have higher plasma 24HC levels, which inversely correlated with age (Grayaa et al., 2018). No changes in the plasma 24HC levels were found in schizophrenia (Chiappelli et al., 2020).

### CSF 24HC in Neurological Diseases

Normally, >99% of brain 24HC is directly fluxed into the systemic circulation, and only <1% is first secreted into the CSF to enter then the systemic circulation (Leoni et al., 2004). Hence, not only the levels of plasma 24HC but also the levels of CSF 24HC could be sensitive to intracerebral changes in cholesterol homeostasis. The latter changes could be a result of cholesterol release from dying cells with subsequent conversion to 24HC by CYP46A1 and/or due to altered CYP46A1 expression or activity. In addition, the levels of plasma and CSF could be affected by dysfunction of the blood-brain and blood-CSF barriers, respectively (Leoni et al., 2003). Indeed, the CSF levels of 24HC were shown to be increased in several conditions.

In AD, the 24HC levels in the CSF appeared to have a better diagnostic power than plasma levels and were even suggested to be a marker of brain health (Leoni et al., 2013b). In most studies, the CSF 24HC was increased in AD (Papassotiropoulos et al., 2002; Schonknecht et al., 2002; Leoni et al., 2006, 2013b; Bjorkhem et al., 2019), possibly due to a sterol release as a result of neurodegeneration (Björkhem and Diczfalusy, 2020) and a lack of confounding contribution of hepatic clearance.

In MS, patients treated with natalizumab, a drug which reduces inflammation and degeneration of the CNS, showed a decrease in the CSF 24HC levels, which was interpreted as an indicator of a reduced neurodegeneration (Novakova et al., 2015).

In PD, an increase in the 24HC levels in the CSF was detected, and 10% of patients even had a correlation between the CSF 24HC and disease duration. Therefore, CSF 24HC was proposed to be of value for following the disease progression (Bjorkhem et al., 2013, Björkhem et al., 2018).

Increased 24HC levels were also detected in several neuroinflammatory diseases (neuroborreliosis, viral meningitis, and Gullian-Barre syndrome) (Leoni et al., 2003, 2004; Bjorkhem et al., 2013, Björkhem et al., 2018).

Thus, a number of brain disorders appeared to have changes in CYP46A1 activity, and these disorders were not only limited to neurodegenerative diseases. Even some conflicting results still need to be clarified, the importance of 24HC quantifications is that they pointed to the diseases which could benefit from CYP46A1 activity modulation and suggested that more neurological conditions should be evaluated for changes in the plasma or CSF 24HC levels.

### CYP46A1 Protein Levels Are Modified in Brain Regions Affected by Neurological Disorders

Not only CYP46A1 activity and concentrations of 24HC vary in the plasma and CSF depending on a specific disease and its stage of progression, but also CYP46A1 protein levels are altered in the brain. In patients with HD as well as Machado-Joseph disease (MJD or Sca3), the levels of CYP46A1 were reduced in the affected brain regions, the striatum in HD and the cerebellum in MJD patients (Boussicault et al., 2016; Nobrega et al., 2019). Interestingly, additional CYP46A1 expression in non-neuronal cells (astrocytes) was documented in the AD- and HD-affected brains, perhaps to compensate for CYP46A1 loss in neurons due to neurodegeneration (Bogdanovic et al., 2001; Brown et al., 2004).

## CYP46A1 Is a Relevant Therapeutic Target in Animal Models

### CYP46A1 Regulation

CYP46A1 does not appear to be regulated at transcriptional levels as shown by experiments on cells transfected with the *CYP46A1* reporter constructs. The *CYP46A1* transcription was found to be insensitive to the major regulatory axes, and only oxidative stress significantly increased the gene transcription (Ohyama et al., 2006). In addition, the specificity (Sp) transcription factors were shown to contribute to the control of the basal *CYP46A1* transcription (Milagre et al., 2008, 2012; Moutinho et al., 2016). Epigenetic mechanisms were shown to alter the *CYP46A1* expression *in vivo* and *in vitro* (Shafaati et al., 2009; Milagre et al., 2010; Nunes et al., 2010).

### Role of CYP46A1 in Neurotransmission and Stress Response

The role of CYP46A1 as an important neuronal stress response factor and the effects of 24HC on neuronal survival were demonstrated (Sodero et al., 2012). Current studies also strongly support the neurotransmission-CYP46A1 activity link (Sodero et al., 2011, 2012; Mast et al., 2017a). *In vitro*, CYP46A1 activity is increased by L-Glu, and 24HC is a potent positive allosteric modulator of the GluN2B subunit of N-methyl-D-aspartate receptors (NMDARs), whose activation is a key mediator of the long-term potentiation (LTP) induction (Paul et al., 2013; Linsenbardt et al., 2014; Emnett et al., 2015; Sun et al., 2016b; Wei et al., 2019). Conversely, *Cyp46a1^–/–^* mice were shown to have reduced activity and function of these receptors, which govern experience-dependent synaptic plasticity (Sun et al., 2016a). Collectively, these results suggest a reciprocal relationship between CYP46A1 activity and neurotransmission: excitatory neurotransmission increases CYP46A1 activity, and increased CYP46A1 activity enhances neurotransmission. This relationship could have important implications for brain plasticity and, interestingly, for both conditions of NMDAR hypofunction and hyperfunction. CYP46A1 activation could be beneficial for AD, HD and other polyglutamine diseases (Scas), in which either brain levels of CYP46A1 or 24HC are decreased (Boussicault et al., 2016; Testa et al., 2016; Nobrega et al., 2019) and, hence, can affect the NMDAR function (Zhou and Sheng, 2013). Conversely, CYP46A1 inhibition could be of therapeutic value in pathological conditions that promote excessive glutamatergic excitation such as those occurring in schizophrenia, epilepsy, and hyperalgesia (Coyle, 2006; Xia et al., 2010; Sun et al., 2016b; Lu et al., 2020). Thus, depending on the context, both upregulation/mimicry of CYP46A1 activity along with 24HC signaling and downregulation/antagonism may have therapeutic potential.

Increased CYP46A1 activity in response to an excitotoxic stress raises the key question of the cause or the consequence of this response. In mice with excessive stimulation of glutamate receptors after a single injection of kainic acid, a loss of membrane cholesterol due to 24-hydroxylation was demonstrated. This cholesterol loss was shown to be a normal response to high excitatory neurotransmission in neurons, and it was suggested to modulate the magnitude of the depolarization-evoked calcium response (Sodero et al., 2012). Consistent with this cause–effect relationship, knockdown of *CYP46A1* prevented Glu-mediated cholesterol loss in cultured neurons (Sodero et al., 2012). In rats exposed for 11 weeks to subneurotoxic doses of L-Glu, the regulation of cholesterol metabolism and transport by chronic Glu exposure was confirmed (Zhang et al., 2020). The brain ratio of 24HC to cholesterol and CYP46A1 expression was increased, and Glu induced an elevation of CYP46A1 and ApoE. These modulations could either be a toxic event or a compensatory change during the chronic excitotoxicity (Zhang et al., 2020). The data obtained are consistent with recent observations in a neonatal model of hypoxic ischemia in mice, in which increased production of 24HC in the injured hemisphere and simultaneous metabolite excretion into the bloodstream were demonstrated (Lu et al., 2021). Enhanced brain cholesterol turnover and upregulation of CYP46A1 were observed. However, further investigations are needed to demonstrate that this is a protective stress response.

### *CYP46a1*^–/^*^–^* and *CYP46A1*^*Tg*^ Mice

*Cyp46a1* knockout did not impair mouse survival, potentially due to the compensatory mechanisms that were in place during development. However, the characterization of *Cyp46a1^–/–^* mice revealed that animals of both sexes had severe cognitive deficits, thus linking CYP46A1, cholesterol turnover in the brain with higher order brain functions (Kotti et al., 2006). Decreased cholesterol biosynthesis in the brain, and thereby decreased protein prenylation by geranylgeraniol (a cholesterol biosynthetic intermediate), were shown to contribute to this link *via* the impairment of the hippocampal LTP (Kotti et al., 2008).

*CYP46A1* transgenic mice were generated as well and demonstrated increased cholesterol elimination from the brain. While no apparent changes were present in young animals, 15-month- old female mice demonstrated improved spatial memory in the Morris water maze (MWM) test and showed increased expression of some of the synaptic proteins, including one of the NMDAR subunits (Maioli et al., 2013).

**FIGURE 2:**
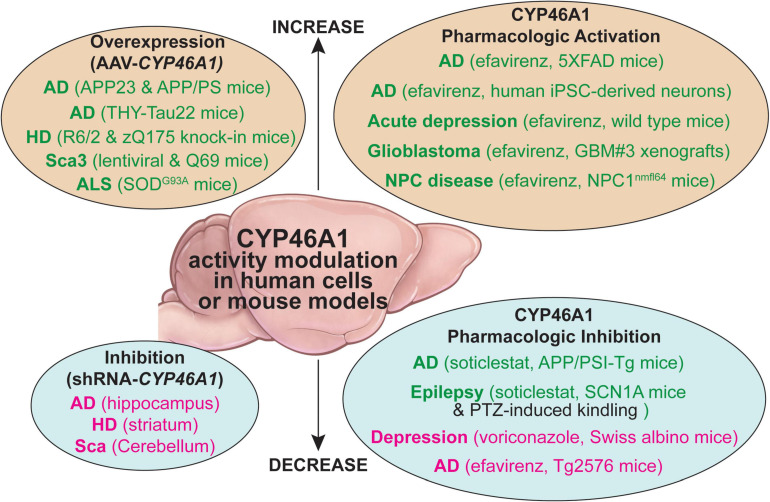
A summary of the CYP46A1 activity modulation by genetic (the AAV-based gene delivery or shRNA inhibition of gene transcription) and pharmacologic means in human cells and mouse models of different brain disorders. AD, Alzheimer’s disease; ALS, amyotrophic lateral sclerosis; HD, Huntington’s disease; NPC, Nieman-Pick type C disease; iPSC, human induced pluripotent stem cells; PTZ, pentylenetetrazol; Sca3, spinocerebellar ataxia 3. The beneficial and detrimental effects of the CYP6A1 activity modulation are indicated in green and magenta fonts, respectively, black font indicates no effects on the studied process.

Altogether, the two *in vivo* models suggested that CYP46A1 could play a key role in memory processes and synaptic plasticity, and that modulating CYP46A1 activity could have therapeutic implications. So far, two major approaches have been tested *in vivo* to investigate CYP46A1 activity modulation—genetic targeting of *CYP46A1* expression and direct CYP46A1 activity modifications by enzyme inhibitors or activators. Both were evaluated in mouse models of different brain diseases (Figure 2).

## Genetic Targeting of CYP46A1 Expression

### CYP46A1 Inhibition Using a Short Hairpin RNA Is Detrimental in Mouse Brain

Decreases of CYP46A1 levels in patients with severe neurodegenerative diseases, like AD, HD, and Scas, and the phenotypic impairment observed in *Cyp46a1^–/–^* mice, prompted studies of potential detrimental consequences of CYP46A1 inhibition in adult mice. This was achieved by injecting an adeno-associated virus (AAV) vector carrying a short hairpin RNA (shRNA) expression cassette specific to the *Cyp46a1* gene.

Hippocampal injection of the AAV-shRNA *Cyp46a1* to wild type mice led to cognitive deficits, hippocampal atrophy, enhanced amyloid β (Aβ) production, abnormal tau phosphorylation, and stress of the ER, all symptoms strongly resembling AD phenotype (Djelti et al., 2015). Injection of the AAV-sh*Cyp46a1* in the APP23 model of AD exacerbated their AD phenotype by increasing the Aβ content, tau phosphorylation, and neuronal loss leading to epileptic seizures (Chali et al., 2015; Djelti et al., 2015). This phenotype was associated with major lipid abnormalities and modifications of the lysosomal compartment. Specifically, accumulation of sphingolipids and increased expression of the enzymes involved in phosphatidylcholine and sphingolipid metabolism after the AAV-sh*CYP46A1* injection were associated with alterations in the lysosomal cargo, accumulation of phagolysosomes, and impairment of endosome-lysosome trafficking (Ayciriex et al., 2017).

In the striatum of wild type mice, the knockdown of the *Cyp46a1* mimicked HD phenotype with spontaneous medium spiny striatal neuron degeneration, motor deficits (Boussicault et al., 2016), and accumulation of endosomes and lysosomes (Nobrega et al., 2019).

### Restoring of CYP46A1 Is Therapeutic in AD, HD, Scas, and ALS Mouse Models

Mouse models of amyloid and tau pathologies, HD, and MJP (Sca3) were shown to have a decrease in the levels of *Cyp46a1* mRNA and protein, thus confirming a decrease in CYP46A1 expression in patients with AD, HD, and Sca3 (Hudry et al., 2010; Burlot et al., 2015; Boussicault et al., 2016; Kacher et al., 2019; Nobrega et al., 2019). The injections of the AAV-*CYP46A1* to the brain of these models were used to increase the P450 expression and positively affect the disease manifestations (Hudry et al., 2010; Burlot et al., 2015; Boussicault et al., 2016; Kacher et al., 2019; Nobrega et al., 2019).

In the AD models of amyloidogenesis (APP23 and APP/PS mice), neuronal overexpression of CYP46A1 before or after the onset of amyloid plaques decreased the brain Aβ deposits and improved animal performance in the MWM test of the investigated APP23 model. This was further confirmed in the very severe APP/PS1 knock-in model in which AAV-CYP46A1 not only decreased amyloid burden but also improved spine density and LTP (Alves et al., 2018). Cerebral delivery of *CYP46A1* was shown to reduce the cleavage of amyloid precursor protein (APP), possibly due to a decreased content of cholesterol and presenilin 1 in lipid rafts; the latter being an important component of the γ-secretase complex, which yields the Aβ peptides (Hudry et al., 2010).

Notably, the therapeutic benefit of CYP46A1 expression was not only observed on the amyloid component of AD but also on the tauopathy. In a model of the AD-like tau pathology (THY-Tau22 mice), the CYP46A1 gene therapy completely restored impaired cholesterol metabolism, rescued cognitive deficits, and mitigated impairments in long-term depression (LTD) as well as spine defects that characterize this model. Tau hyperphosphorylation and the associated gliosis were not reduced, but the persistence of glial cells could be in line with the role of glial cells in the clearance of misfolded protein accumulation in AD (Burlot et al., 2015).

Defects of brain cholesterol metabolism are particularly well documented in HD and are associated with increased content of membrane cholesterol and a decreased production of cholesterol due to a reduction in cholesterol biosynthesis (Karasinska and Hayden, 2011; Alves et al., 2018). In R6/2 and zQ175 knock-in mice, *CYP46A1* delivery into the striatum rescued the cholesterol synthesis pathway, improved motor function, and decreased neuronal atrophy. Importantly, the AAV-*CYP46A1* administration significantly decreased the number and area of the polyglutamine (polyQ)-mutant huntingtin aggregates in both models to 40–50% (Boussicault et al., 2016; Kacher et al., 2019). These studies allowed major insights into the mechanisms of therapeutic effects of CYP46A1 *in vivo* as the striatal transcriptomic profile of zQ175 mice was improved by the *CYP46A1* injections. CYP46A1 increased expression of genes implicated in lipid metabolism, synaptic transmission, autophagy, innate immunity, and DNA repair. Experimental evidence was presented that these modifications were translated into improvements of synaptic activity and connectivity along with the activation of the proteasome and autophagy machineries, which participate in the clearance of the mutant polyQ-huntingtin aggregates (Kacher et al., 2019). In particular, it was shown that the maturation of autophagosomes was improved. It was also further confirmed that CYP46A1 expression is protective against NMDAR-mediated excitotoxicity in two different HD neuronal cell models and helps to reduce neuronal cholesterol content in lipid raft extracts (Boussicault et al., 2018).

MJD (Sca3), a disease characterized by the accumulation of polyQ-ataxin-3, is the most prevalent form of ataxia worldwide. The CYP46A1 protein level is decreased in the cerebellum of MJD patients (Nobrega et al., 2019). To investigate potential therapeutic consequences of restoring CYP46A1 expression, two models (lentiviral and Q69) of Sca3 were tested as decreased expression of CYP46A1 was confirmed in the brain of Sca3 mice. In the lentiviral-based model, the striatal administration of the AAV-*CYP46A* reduced the accumulation of the mutant ataxin-3 aggregates, a hallmark of Sca3, and preserved neuronal markers (Nobrega et al., 2019). In the severe transgenic Sca3 Q69 model, cerebellar delivery of *CYP46A1* was strongly neuroprotective in adult mice through the significant decrease of the ataxin-3 aggregation, the alleviation of motor impairments, and the improvement of the Sca3-associated neuropathology (preservation of Purkinje cells and of the cerebellum volume). *CYP46A1* activated autophagic clearance of ataxin-3 (Nobrega et al., 2019).

The role of the cholesterol pathway and CYP46A1 activity were recently investigated in ALS. Cholesterol metabolism seems to play a major role in neuromuscular junctions, synaptic vesicle cycle, neurotransmitter release, and synaptic integrity (Krivoi and Petrov, 2019). The effect of exogenous 24HC on neuromuscular transmission in the diaphragm of SOD^*G9*3A^ mice, a model of ALS, was evaluated (Mukhutdinova et al., 2018). 24HC was found to suppress the exocytotic release of neurotransmitter in response to intense activity *via* the NO/lipid raft-dependent pathway. Also, 24HC increased the staining of the neuromuscular junction membranes with CTxB (a lipid raft marker), indicating an increase in membrane ordering that likely attenuated NO production. Moreover, treatment with the raft-disrupting agents (methyl-β-cyclodextrin or sphingomyelinase) markedly suppressed the effect of 24HC on both lipid raft integrity and activity-induced NO production. These results were consistent with increased sphingomyelinase gene transcription observed after CYP46A1 inhibition in the brain of normal mice (Ayciriex et al., 2017). Given that the disturbance of cholesterol-rich microdomains at the neuromuscular junctions might occur as a result of muscle disease and affect synaptic transmission (Gil et al., 2006; Petrov et al., 2017), the potential membrane ordering effect of 24HC might protect neuromuscular junctions (Mukhutdinova et al., 2018).

Beneficial role of CYP46A1 in ALS was confirmed in SOD1^*G*93A^ mice. A single intravenous administration of AAV-*CYP46A1* at the presymptomatic or symptomatic stage improved the severe phenotype characterized by a rapid decrease in muscular strength and motor dysfunction leading to progressive paralysis at 3 months. A significant and prolonged motor rescue of animals treated at the preventive or symptomatic stages was observed and improved survival. Clinical improvement was associated with preservations of motoneurons in the spinal cord, the muscular fiber structure, and neuromuscular junctions (Piguet et al., 2019; Wurtz et al., 2020).

Collectively, the AAV-*CYP46A1* delivery to animal models of AD, HD, Sca3, and ALS enabled major insights into the role of the CYP46A1 defect in neurodegenerative diseases and CYP46A1 mechanisms of therapeutic action. These extensive studies provided strong support for CYP46A1 as a therapeutic target and suggested that a one-time *CYP46A1* delivery approach could be translatable to human patients. Feasibility and safety proof-of-concept experiments in non-human primates are ongoing in preparation for the submission of a first clinical application in human patients (Piguet et al., 2020).

## Pharmacologic Targeting of CYP46A1 Activity

Screening of the marketed drugs for the effect on activity of purified CYP46A1 *in vitro* led to the identification of both CYP46A1 inhibitors and activators (Mast et al., 2008, 2012) and gave impetus to *in vivo* testing of the identified modulators. Results from these studies have shed light on the CYP46A1 activity effects in different pathophysiological conditions.

### Consequences of Pharmacological Inhibition of CYP46A1

The antifungal medicine voriconazole, a CYP46A1 inhibitor, was the first enzyme modulator that was tested *in vivo* on wild type mice. Intraperitoneal voriconazole injections (once a day for 5 consecutive days) of a clinically relevant drug dose (60 mg/kg of body weight) were used and resulted in reduced brain 24HC levels. This reduction led in turn to a compensatory decrease in the brain cholesterol biosynthesis; hence, the brain cholesterol levels remained unchanged (Shafaati et al., 2010). Thus, it was revealed that *in vivo*, pharmacologic inhibition of CYP46A1 could be achieved within a relatively short time and does not affect the tight coupling between the brain cholesterol elimination and brain cholesterol biosynthesis. Voriconazole was then evaluated on a mouse model of depression and recently on hippocampal slices of rats (Patel et al., 2017; Popiolek et al., 2020). In mice with depression, the CYP46A1-inhibiting dose of voriconazole (75 mg/kg of body weight per day) had a pro-depressive effect and decreased the brain serotonin levels (Patel et al., 2017). In hippocampal slices of rats, voriconazole inhibited LTD at a 3 μM concentration (Popiolek et al., 2020).

Due to an interest in CYP46A1 inhibitors, new compounds have been developed for commercialization, and several of them were assessed *in vivo*. Two new CYP46A1 inhibitors were evaluated at different doses and post-treatment times on wild type mice, and one of them was shown to decrease the brain 24HC levels as early as 4 h post-treatment. In hippocampal slices, these inhibitors ablated LTD at 1 or 10 μM concentrations, a comparable effect to that of voriconazole (3 μM) (Popiolek et al., 2020).

In a different investigation, a novel CYP46A1 inhibitor called soticlestat (also known as TAK-935 and OV935) was administered to the AD model of amyloidogenesis (APP/PS1-Tg mice) and evaluated for pharmacokinetics, pharmacodynamics, and functional effects. In only 8 h (the first measured time point), soticlestat inhibited CYP46A1 after a single oral dose of 10 mg/kg of body weight, whereas the three daily administrations reduced the brain 24HC levels to a steady state (Nishi et al., 2020). An 8-week treatment of young APP/PS1-Tg mice with soticlestat (10 mg/kg of body weight) starting at the age of 7 weeks increased animal survival from 16 to 28 in the groups of 30 animals. A 2-week treatment of APP/PS1-Tg mice with the same dose of soticlestat followed by a 100 mM KCl hippocampal perfusion to induce the hyperexcitability phenotype suppressed the elevation of extracellular Glu and reduced seizure-like behaviors. In another experiment, a 2-week treatment of 3-month old APP/PS1-Tg mice did not alter the hippocampal levels of the Aβ42 peptide and did not have notable effects on motor coordination or spontaneous locomotor activity (Nishi et al., 2020). Besides APP/PS1-Tg mice, soticlestat was assessed on mouse models of epilepsy (*SCN1A* mice and pentylenetetrazol-induced kindling) and was shown to decrease seizure burden in the former but not the latter (Bialer et al., 2018), although full papers describing these findings remain to be published. Based on these animal studies, soticlestat was advanced to a clinical trial, which will be presented in the next section.

The ^11^C-labeled form of soticlestat was synthesized for use in positron emission topography (Chen et al., 2020). Yet, the drug characterization in this study yielded different results when compared to those of the developers of this compound (Nishi et al., 2020). ^11^C-soticlestat was found to have low uptake by the brain and a marginal CYP46A1 specificity (Chen et al., 2020). Furthermore, experiments with purified recombinant human CYP46A1 and subsequent 24HC quantifications by isotope-dilution gas chromatography-mass spectrometry, a highly accurate method, did not confirm tight soticlestat binding to P450 as indicated by a high *K*_*i*_ value of 7.3 μM (Chen et al., 2020). This is in contrast to the reported 4.5 nM *IC*_50_ determined in cultured cells transfected with *CYP46A1* using 24HC detection by thin layer chromatography (Nishi et al., 2020). Apparently, additional soticlestat characterizations are needed to ascertain its binding specificity for CYP46A1.

### CYP46A1 Activation

Pharmaceuticals that activate CYP46A1 *in vitro* were found during screening of the FDA-approved drugs for CYP46A1 inhibition (Mast et al., 2008, 2012). This was particularly exciting because not every enzyme could be activated, and only very few drugs on the market act as enzyme activators rather than inhibitors (Blum et al., 2011; Haberle, 2011; Matschinsky et al., 2011). A CYP46A1 activator and anti-HIV drug efavirenz (EFV) was chosen for subsequent studies in mice, which proved to be a challenge as many drug doses and treatment durations had to be tested to identify the treatment paradigm that activated CYP46A1 *in vivo* (Mast et al., 2014). Ultimately, the CYP46A1 activating dose was identified and appeared to be very small (0.1 mg/kg of body weight per day delivered in drinking water), ∼86-times lower than that given to HIV-positive subjects to keep their viral load low (∼8.6 mg/kg of body weight per day or 600 mg/day). Increasing the EFV daily dose to > 0.2 mg/kg of body weight inhibited CYP46A1 and brain cholesterol 24-hydroxylation, thus indicating a narrow therapeutic window for CYP46A1 activation by EFV. Mouse treatment by EFV had to last at least 4 weeks for the brain sterol levels to reach a new steady state, at which point the content of 24HC as well as lathosterol and desmosterol [cholesterol precursors and markers of cholesterol biosynthesis in the neurons and astrocytes, respectively (Nieweg et al., 2009)] were increased but the cholesterol levels remained unchanged. This new steady state indicated a coupling between increased cholesterol elimination and biosynthesis in the brain to keep the brain cholesterol at normal levels (Mast et al., 2014). Mechanistically, EFV was found to be the CYP46A1 allosteric activator, which bound only to the allosteric site when used at small concentrations and to both allosteric and active sites when used at high concentrations (Mast et al., 2014; Anderson et al., 2016).

The CYP46A1 activating EFV dose of 0.1 mg/kg of body weight per day was next tested on the AD model of rapid amyloidogenesis (5XFAD mice) in two treatment paradigms. In the first, mice were started on EFV at 1 month of age before the Aβ plaque appearance and continued for 8 months until 9 months of age (Mast et al., 2017c). In the second treatment paradigm, the drug administration began at 3 months of age, after the amyloid plaques developed, and continued for 6 months until 9 months of age (Petrov et al., 2019a). In both paradigms, CYP46A1 was activated, the brain cholesterol turnover was enhanced, and mouse performance in the MWM test was improved (Mast et al., 2017c; Petrov et al., 2019a). The common general effects included changes in gene expression and protein phosphorylation (also observed in *Cyp46a1^–/–^* mice), which encompassed targets from different pathways and processes (e.g., APP processing, inflammation, immune response, autophagy, ubiquitin-proteasome systems, hypoxia, apoptosis, glucocorticoid-related stress, synaptic function, neurite growth and Ca^2+^-, small GTPase, and catenin signaling) (Mast et al., 2017b, 2020a; Petrov et al., 2019b). Changes in the Aβ levels, astrocyte and microglia activation, and expression of essential synaptic proteins were treatment paradigm-specific with both age (or initial Aβ load) and treatment duration appearing to determine, in part, the outcome of treatment (Mast et al., 2017c; Petrov et al., 2019a). Notably, when EFV was tested in a different study using the AD model of cerebral amyloidosis (Tg2576 mice) and the CYP46A1-inhibiting dose (15 mg/kg of body weight for 10 days), the drug increased the brain Aβ load (Brown et al., 2014).

In a human model of AD (induced pluripotent stem cell-derived neurons and astrocytes), EFV treatment (10 μM) reduced aberrant accumulation of phosphorylated tau in the neurons, which was independent of APP and Aβ. Importantly, EFV was well tolerated by astrocytes and was found to reduce the amount of esterified cholesterol in neurons, thereby leading to an increase in proteasomal activity and proteasomal degradation of phosphorylated tau (van der Kant et al., 2019a,b).

Besides models of AD, EFV was tested on mouse models of acute depression, glioblastoma, Nieman-Pick type C disease, and prion-infected mice (Patel et al., 2017; Han et al., 2019; Mitroi et al., 2019; Ali et al., 2021). These treatments demonstrated that therapeutic benefits of CYP46A1 activation may not be limited to AD. In stressed mice, EFV showed anti-depressive effects and increased the brain serotonin levels (Patel et al., 2017). In mice bearing intracranial tumors (the LN229 and GBM#P3 orthotopic xenografts), EFV treatment prolonged animal survival and inhibited tumor growth in the GBM#P3 model. Mechanistically, EFV administration was shown to affect the tumor cholesterol homeostasis by regulating the activity of the two important transcription factors–liver X receptors (LXRs), for which 24HC is a ligand (Janowski et al., 1996), and sterol regulatory element-binding protein 1 (SREBP1), a sensor of cellular cholesterol and oxysterol levels (Goldstein et al., 2006). In addition, the treatment increased the levels of cleaved poly (ADP-ribose) polymerase (PARP, an apoptosis marker) and decreased the levels of proliferating cell nuclear antigen (PCNA, a proliferation marker) as well as SRY (sex determining region Y)-box transcription factor 2 (SOX2, a stemness marker) (Han et al., 2019). In a mouse model of Nieman-Pick type C disease (*NPC1^*nmfl64*^* mice), EFV treatment extended the mouse life span by 30%, normalized synaptic levels of cholesterol and LTP, and improved performance in behavioral tests as well as the lysosomal phenotype (Mitroi et al., 2019). In prion-infected mice, EFV administration significantly mitigated the propagation of the infectious isoform of the cellular prion protein while preserving physiological cellular prion protein and lipid raft integrity. Also, drug treatment significantly prolonged the lifespan of animals (Ali et al., 2021).

To discriminate between CYP46A1-dependent and independent effects of EFV treatment, *Cyp46a1^–/–^*5XFAD mice were recently generated and treated along with 5XFAD animals with EFV (0.1 mg/kg body weight per day) for 6 months, from 3 to 9 months of age (Mast et al., 2020a). A comparison of these two lines vs. their corresponding controls indicated that the CYP46A1-dependent EFV effects include changes in the levels of brain sterols, steroid hormones, proteins such as glial fibrillary acidic protein (GFAP, a marker of astrocyte activation), ionized calcium binding adaptor molecule 1 (Iba1, a marker of microglia activation), Munc13-1, post synaptic density-95 (PSD-95), gephyrin, synaptophysin, and synapsin-1, as well as genes involved in neuroprotection, neurogenesis, synaptic function, inflammation, oxidative stress, and apoptosis. The CYP46A1-independent EFV effects included the transcription of genes from cholinergic, monoaminergic, and peptidergic neurotransmission, the homeostasis of sulfated steroids, steroidogenesis, and vitamin D_3_ activation. Apparently, even at a small dose, EFV acted as a transcriptional regulator, yet this regulation did not appear to lead to functional effects. This study further confirmed that CYP46A1 is a key enzyme for cholesterol homeostasis in the brain, and that the therapeutic EFV effects on 5XFAD mice are likely mostly realized *via* CYP46A1 activation (Mast et al., 2020a).

Of pertinence to the CYP46A1-independent EFV effects is a study showing increased somatic *APP* recombination in neurons from individuals with AD as compared to neurons from individuals who lacked the disease (Lee et al., 2018). This recombination was shown to depend, among other processes, on reverse transcriptase activity and was linked to AD, which is known to be rare in HIV-positive individuals 65 years of age and older (Turner et al., 2016). This work supported testing of the anti-HIV reverse transcriptase inhibitors in AD patients (Lee et al., 2018) and suggested that a potential CYP46A1-independent EFV effect could be of benefit for treatment of AD.

The search for CYP46A1 activators continues. The goal is to identify compounds that have both a broader therapeutic window and a higher potency for CYP46A1 activation than EFV. These properties should enhance the potentially beneficial effects of CYP46A1 activation and lower the possibility of CYP46A1 inhibition at high EFV doses (Mast et al., 2020b). Eight EFV-related compounds were evaluated *in vitro* on purified CYP46A1, and one (8,14-dihydroxyEFV) activated CYP46A1 1.5-times stronger than EFV. Importantly, 8,14-dihydroxyEFV did not inhibit CYP46A1 at the range of the concentrations (60–100 μM) that were already inhibiting for EFV. Remarkably, 8,14-dihydroxyEFV is an initial product of EFV clearance by the liver, thus suggesting that not only EFV but also its metabolites could activate CYP46A1 *in vivo*. Furthermore, 8,14-dihydroxyEFV was used as a racemic mixture but not as a pure *S* enantiomer, like EFV, and evidence was obtained that CYP46A1 could be activated by both, *S* and *R* EFV metabolites. This finding pointed to replacing *S*-EFV with racemic EFV as an approach to avoid possible EFV toxicity at high doses (Mast et al., 2020b). Experiments on 5XFAD mice are in progress to evaluate the effects of racemic 8,14-dihydroxyEFV.

## CYP46A1 as a Therapeutic Target in Human Diseases

Preclinical proofs of concept have provided strong support for CYP46A1 being a viable therapeutic target and justified the initiation of first clinical trials. Currently, one trial (NCT03650452^1^) has already been completed on July 20, 2020, and another (NCT03706885, see text footnote 1) is still on-going.

The NCT03650452 clinical trial (called ELEKTRA) was a phase 2 study to evaluate the efficacy, safety, and tolerability of soticlestat as a CYP46A1 inhibitor in pediatric patients with Dravet syndrome and Lennox Gastaut syndrome. In these diseases with excessive glutamatergic excitation, CYP46A1 inhibition was hypothesized to negatively modulate NMDARs and be of therapeutic value. One hundred forty-one participants were enrolled and 126 completed the study, which had two arms, experimental and placebo. Both arms had 2 periods: the screening to establish the baseline seizure frequency; and the treatment period, which consisted of an 8-week dose optimization phase followed by a 12-week maintenance period. The primary endpoint was% change from baseline in frequency of all seizures per 28 days in the treatment vs. placebo arms during the maintenance period. No results have yet been published. A press release from Takeda Pharmaceutical Company Limited and Ovid Therapeutics^2^ ^3^ stated that the study achieved its primary endpoint. For the 12-week maintenance period, the median changes from the baseline in seizure frequency in the combined Dravet and Lennox Gastaut syndrome cohorts were −27.8 and + 3.1% in the treatment and placebo arms, respectively (*P* = 0.0007). For the full 20-week treatment period, the corresponding numbers were −29.8 and 0.0% (*P* = 0.0024). When the data for the latter were broken down by the cohorts, the changes for the Dravet syndrome cohort (convulsive seizure frequency) were −33.8 and + 7.0% (*P* = 0.0007) and for the Lennox Gastaut syndrome cohort (drop seizure frequency) were −20.6 and −6.0% (*P* = 0.1279). There was a reduction of plasma 24HC levels with soticlestat treatment vs. placebo. All patients who completed the ELEKTRA study elected to enroll into the ENDYMION open-label extension study. The primary objectives of the ENDYMION are to assess the long-term safety and tolerability of soticlestat over a 4 year treatment in patients with rare epilepsies and to evaluate the effect of soticlestat on seizure frequency over time (see text footnote 3). Thus, the ELEKTRA trial supports that CYP46A1 inhibition could reduce seizure frequency in children with certain epileptic disorders.

While inhibition of CYP46A1 was proposed to be beneficial in very specific diseases with excessive glutamatergic excitation, a large number of neurodegenerative conditions are associated with glutamatergic hypofunction. In such situations converging proofs-of-concept mentioned above were made in animal models that set the basis for therapeutic intervention aiming to activate or deliver CYP46A1 to the affected brain regions. Reproducible demonstrations in mouse models of AD of beneficial effects of the CYP46A1 expression increase or CYP46A1 pharmacologic activation justified the safety and tolerability phase 1 study of EFV (Sustiva) in patients with mild cognitive impairment due to AD (NCT03706885 trial called EPAD). At a 600 mg per day dose given to HIV-positive subjects, EFV can have some adverse effects (Apostolova et al., 2015, 2017) and likely inhibits CYP46A1. Therefore, lower EFV doses are investigated for CYP46A1 activation and safety in the geriatric population. Specifically, the study has three arms, two treatment and one placebo, with participants in the treatment arms receiving 50 and 200 mg of EFV daily for 20 weeks. The trial’s primary endpoint is to identify EFV doses that engage CYP46A1, as assessed by changes in the plasma 24HC levels. In addition, participants are genotyped for the APOE isoform status (E2, E3, or E4) and the presence of SNPs rs754203 and rs3745274 in *CYP46A1* and *CYP2B6*, respectively. This is done because the APOE status and *CYP46A1* SNP may affect CYP46A1 activity, whereas the *CYP2B6* SNP can increase the plasma EFV concentrations because CYP2B6 is the major enzyme metabolizing EFV (Ward et al., 2003). The trial is planned to be completed in summer of 2021 and will provide insight into whether the investigated EFV doses activate CYP46A1 in human brain or if EFV doses should be lowered to accomplish this goal.

## How Altered CYP46A1 Activity Can Affect Multiple Cellular Processes in Various Brain Diseases

The primary and proven role of CYP46A1 is to catalyze cholesterol 24-hydroxylation, the rate-limiting step in cholesterol removal from the brain and the reaction that controls the brain cholesterol turnover (Lutjohann et al., 1996; Bjorkhem et al., 1998; Lund et al., 1999, 2003). Yet, numerous studies of the biological effects of CYP46A1 activity modulation suggest that the roles of CYP46A1 in the brain are much broader than originally thought and extend beyond those in cholesterol elimination. Collectively, CYP46A1 activity modulation was shown to alter processes such as synaptic plasticity; gene expression; protein phosphorylation; apoptosis; inflammation; immune response; proteasome, ubiquitin, and autophagy systems; endosomes, lysosomes, and ER; as well as signaling *via* different receptors. In addition, CYP46A1 was demonstrated to be a major neuronal stress response factor in conditions like aging, excitatory neurotransmission, and accumulation of reactive oxygen species by regulating the TrkB/PI3K/Akt pro-survival pathway (Sodero et al., 2011).

Altogether these results raise the question of general unifying mechanisms for the multiple CYP46A1 activity effects. In addressing this question, the four types (individual or combined) of the CYP46A1 activity effects should be considered: (1) on total brain cholesterol levels and cholesterol distribution in the membrane compartments; (2) on total brain levels of 24HC; (3) on levels of the precursors in the mevalonate pathway (e.g., desmosterol and geranylgeraniol) that play key roles in neuronal function (Moutinho et al., 2017); and (4) on the rate of the brain cholesterol turnover (reviewed in Petrov and Pikuleva, 2019).

**FIGURE 3:**
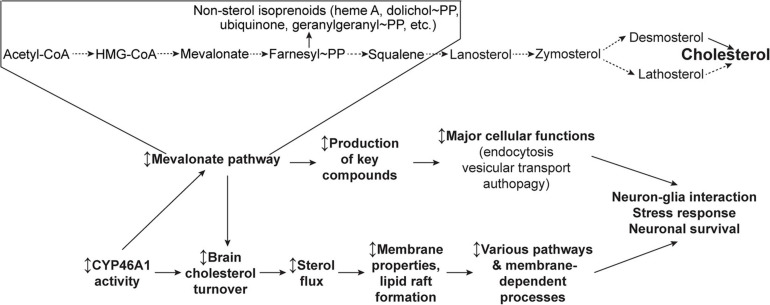
Schematic representation of the two general CYP46A1 mechanisms of action—*via* the mevalonate pathway and sterol flux. Modulation (the ↕ up-down arrow) of CYP46A1 activity affects the mevalonate pathway (or the rate of cholesterol biosynthesis), a critical metabolic circuitry which plays key roles in multiple cellular processes by synthesizing the non-sterol isoprenoids and sterol cholesterol precursors. Modulation of CYP46A1 activity also alters the brain cholesterol turnover and sterol flux through the plasma membranes. The latter in turn affects membrane properties and thereby membrane-dependent events. See main text for details. Dashed arrows indicate multiple steps.

Herein we will focus on how, by both activating the cholesterol synthesis (the mevalonate pathway) and ensuring the cholesterol turnover (or sterol flux), CYP46A1 may exert broad effects on neuronal functions and survival (Figure 3).

### The Role of the Mevalonate Pathway

Cholesterol biosynthesis, called the mevalonate pathway, leads to the production of the key compounds, besides cholesterol, that are involved in diverse cellular processes such as transcription (isopentenyl tRNAs), protein N-glycosylation (dolichol phosphate), protein prenylation (farnesylation and geranylgeranylation), and mitochondrial electron transport (ubiquinone) (Figure 3). Although cholesterol biosynthesis mostly occurs in astrocytes in adults, neurons keep the mevalonate pathway active, even with sustained cholesterol supply from the aforementioned astrocytes, to ensure the production of these essential compounds (Moutinho et al., 2017). Isoprenoids from the mevalonate pathway were shown to act as anchors for membrane association after being covalently bound to proteins like most of the small guanosine triphosphate-binding proteins, which are critical to neuronal cell function. The mevalonate pathway also influences cell size, growth, and proteostasis by regulating basal autophagic flux through geranylgeranylation of the small GTPase RAB11 (Moutinho et al., 2017). Accordingly, the control of autophagy by the mevalonate pathway has potential implications for inflammation and neurodegenerative diseases (Miettinen and Bjorklund, 2016). This role on autophagy has a major impact on a number of neurodegenerative diseases in which the accumulation of misfolded proteins is critical in disease phenotype.

### The Sterol Flux Hypothesis

The sterol flux hypothesis (Figure 3) was put forward and tested based on the characterizations of EFV-treated vs. control 5XFAD mice and *Cyp46a1^–/–^* vs. wild type mice, which had improved and impaired performance, respectively, in behavioral tests and changes in the brain phosphoproteome (Mast et al., 2017b,c; Petrov and Pikuleva, 2019; Petrov et al., 2019b). These animals also had an increase (EFV-treated 5XFAD mice) and decrease (*Cyp46a1^–/–^*) in their brain cholesterol turnover (Mast et al., 2017c; Petrov and Pikuleva, 2019), and hence, increased and decreased, respectively, sterol fluxes through the plasma membranes. Accordingly, it was hypothesized that it could be the sterol flux rates that mainly mediate the CYP46A1 activity effects as they alter the properties of the plasma membranes and/or membrane lipid rafts that serve as the major platforms for signal transduction and targeted protein trafficking (Petrov and Pikuleva, 2019; Petrov et al., 2019b, 2020). The latter would be consistent with the modifications of the lipid raft cholesterol content after CYP46A1 modulation, which was demonstrated earlier in several models (Hudry et al., 2010; Djelti et al., 2015; Boussicault et al., 2018). Accordingly, the membrane and/or lipid raft effects of the CYP46A1 activity modulation could then trigger changes in various brain processes including synaptic transmission and protein phosphorylation. The former is a membrane-dependent process (Jahn and Sudhof, 1994), and the latter relies on the activity of protein kinases and phosphatases, many of which reside in, or are associated with, the membranes and lipid rafts (Antal and Newton, 2013; Prakash, 2020). These sterol flux effects could be similar to those of the altered membrane cholesterol content as they are known to affect the physico-chemical properties of the membranes, the formation of lipid rafts, and the conformation and membrane distribution of integral membrane proteins (Simons and Ehehalt, 2002; Pike, 2003; Yang et al., 2016).

To test the sterol flux hypothesis, synaptosomal factions from the brain of EFV-treated and control 5XFAD mice, *Cyp46a1^–/–^* and wild type mice as well as B6SJL mice, the background strain for 5XFAD animals, were prepared and characterized (Petrov et al., 2020). In EFV-treated *vs* control 5XFAD mice, these fractions had increases in cholesterol accessibility, membrane ordering, resistance to osmotic stress, thickness of the synaptic membranes, total Glu content, and ability to release Glu in response to mild stimulation. Conversely, synaptosomal fractions from *Cyp46a1^–/–^* mice had opposite changes in all these properties consistent with the opposite change in the sterol flux rate as compared to EFV-treated 5XFAD mice. Furthermore, incubations of synaptosomal fractions with the inhibitors of glycogen synthase kinase 3, cyclin-dependent kinase 5, protein phosphatase 1/2A, and protein phosphatase 2B revealed that increased sterol flux in EFV-treated *vs* control 5XFAD mice affected the ability of all four enzymes to modulate Glu release. In contrast, in *Cyp46a1^–/–^* vs. wild type mice, decreased sterol flux altered the ability of only cyclin-dependent kinase 5 and protein phosphatase 2B to regulate the glutamate release (Petrov et al., 2020). Thus, evidence was obtained in support of the cytochrome P450 46A1-mediated sterol flux as an important contributor to the fundamental properties of the membranes and thereby protein phosphorylation and synaptic transmission. An explanation was provided for how one enzyme, CYP46A1, can affect multiple pathways and processes in the brain and serve as a common potential target for various brain disorders. Additional testing is required to further prove or disprove the sterol flux hypothesis.

## Pressing Questions and How to Address Them

Complementary expertise and efforts of many laboratories throughout the world contributed to our current knowledge of CYP46A1 and suggested that this P450 is of translational value. Hence, the future directions in the CYP46A1 research should include those related to the CYP46A1 mechanism of action as well as its therapeutic applications as exemplified by the questions below.

1. How can CYP46A1 affect multiple brain processes and be associated with different brain diseases? Most of the CYP46A1 activity effects could be explained by the sterol flux hypothesis and the production of the intermediates in the mevalonate pathway. Nevertheless, other unifying hypotheses are needed to fully understand the brain significance of CYP46A1. These hypotheses should certainly consider the biological activity of 24HC, a signaling molecule, which binds to different receptors and other important proteins. In parallel, testing of the sterol flux hypothesis and the role of the biosynthetic cholesterol precursors should continue to identify more processes and proteins in the brain as well as in the plasma membranes and/or lipid rafts that depend on cholesterol input and turnover.
2. What is the CYP46A1 role *in vivo* in non-neuronal cells? What is the reason for ectopic CYP46A1 expression in brain astrocytes in AD (Bogdanovic et al., 2001; Brown et al., 2004), in microglia in a mouse model of traumatic brain injury (Cartagena et al., 2008), and in macrophages in a mouse model of MS (Teunissen et al., 2007)? Could CYP46A1 be expressed in the plasma membranes upon excessive stimulation of glutamate receptors in mice (Sodero et al., 2012) and could it be catalytically active if its activity requires a redox partner (cytochrome P450 oxidoreductase) and a source of reducing equivalents (NADPH)? Specific experiments should be designed and conducted to address these important questions and further advance our knowledge of cellular and subcellular CYP46A1 effects.
3. Why are the CYP46A1 levels decreased in certain diseases? Is increased CYP46A1 expression, as evidenced at an early stage of hypoxia, a protective mechanism or a cause of tissue damage (Lu et al., 2018)? Impaired transcription is a common feature of many neurodegenerative diseases, as misfolded protein accumulation in AD, HD, PD, or ALS severely affects the transcription of multiple genes (Yang and Hu, 2016). Because CYP46A1 is a key enzyme of cholesterol balance and an essential neuronal stress response factor, the defect of its gene transcription could lead to severe dysfunction as observed in HD or Sca3 and after *in vivo* inhibition in different brain regions through shRNAs. Increased CYP46A1 expression at an early stage of hypoxia could thus be viewed as a protective mechanism that could be enhanced to potentially improve or accelerate recovery.
4. How to use CYP46A1 delivery/activation as a therapeutic strategy in humans? What are the CYP46A1 activating doses of EFV in humans? In diseases with decreased CYP46A1 protein levels, the beneficial effects of EFV might be limited. Combining *CYP46A1* delivery and EFV activation could therefore represent a relevant option.
5. Does only EFV activate CYP46A1 *in vivo*, or do some of its metabolites also act as CYP46A1 activators, potentially better than EFV? Clinical trials are required to address these questions, and EFV pills need to be manufactured with a smaller drug content than that which is currently available (50, 200, and 600 mg) to test a much broader range of EFV doses.

## Author Contributions

IP and NC wrote the manuscript and are accountable for the content of the work. Both authors contributed to the article and approved the submitted version.

## Conflict of Interest

The authors declare that the research was conducted in the absence of any commercial or financial relationships that could be construed as a potential conflict of interest.

## Footnotes

1 ClinicalTrials.gov

2 https://ovidrx.com/science/clinical-studies/

3 https://www.businesswire.com/news/home/20200825005303/en/

## Abbreviations

24HC: 24-hydroxycholesterol
AAV: adeno-associated virus
AD: Alzheimer’s disease
A β: amyloid β
ALS: amyotrophic lateral sclerosis
APP: amyloid precursor protein
CSF: cerebrospinal fluid
EFV: efavirenz
ER: endoplasmic reticulum
Glu: glutamate
HD: Huntington’s disease
LTD: long-term depression
LTP: long-term potentiation
MJD: Machao-Joseph disease
MWM: Morris water maze
NMDAR: N-methyl -D-aspartate receptor
polyQ: polyglutamine
Sca: spinocerebellar ataxia
shRNA: short hairpin RNA.
